# A New Wrist-Worn Tool Supporting the Diagnosis of Parkinsonian Motor Syndromes

**DOI:** 10.3390/s24061965

**Published:** 2024-03-19

**Authors:** Luigi Battista, Antonietta Romaniello

**Affiliations:** 1 Faculty of Medicine and Surgery, Catholic University of the Sacred Heart, Sede di Potenza, 85100 Potenza, Italy; 2 R&D Department, Biomedical Lab SRL, 85100 Potenza, Italy; 3 Neurology Unit, Department of Neurosurgery, Hospital of Potenza “San Carlo”, Via Potito Petrone, 85100 Potenza, Italy

**Keywords:** Parkinson’s disease, accelerometer, wearable technology, digital health, digital biomarkers

## Abstract

To date, clinical expert opinion is the gold standard diagnostic technique for Parkinson’s disease (PD), and continuous monitoring is a promising candidate marker. This study assesses the feasibility and performance of a new wearable tool for supporting the diagnosis of Parkinsonian motor syndromes. The proposed method is based on the use of a wrist-worn measuring system, the execution of a passive, continuous recording session, and a computation of two digital biomarkers (i.e., motor activity and rest tremor index). Based on the execution of some motor tests, a second step is provided for the confirmation of the results of passive recording. In this study, fifty-nine early PD patients and forty-one healthy controls were recruited. The results of this study show that: (a) motor activity was higher in controls than in PD with slight tremors at rest and did not significantly differ between controls and PD with mild-to-moderate tremor rest; (b) the tremor index was smaller in controls than in PD with mild-to-moderate tremor rest and did not significantly differ between controls and PD patients with slight tremor rest; (c) the combination of the said two motor parameters improved the performances in differentiating controls from PD. These preliminary findings demonstrate that the combination of said two digital biomarkers allowed us to differentiate controls from early PD.

## 1. Introduction

Parkinson’s disease (PD) is the second most common neurodegenerative disorder, affecting an estimated 10 million people worldwide. Typically, PD emerges between the ages of 55 and 65 years, occurring in 1–2% of individuals over 60, with prevalence rising to 3.5% among those aged 85–89 [1,2,3].

In accordance with the Movement Disorder Society (MDS) Clinical Diagnostic Criteria for PD, until definitive validated diagnostic markers become available, clinical expert opinion remains the gold standard diagnostic approach. Diagnosis of PD involves a two-step process: firstly, confirming the presence of bradykinesia alongside either rest tremor, rigidity, or both; subsequently, determining whether the Parkinsonian motor syndrome is attributable to PD [4].

For the initial step, the MDS Criteria define the examination of all cardinal manifestations (bradykinesia, in combination with rest tremor, rigidity, or both), as outlined in the Motor Examination section (Part 3) of the Unified Parkinson’s Disease Rating Scale (MDS-UPDRS) [4,5]. While the MDS-UPDRS assesses PD severity, it does not define the condition, hence no single cutoff score on the MDS-UPDRS items should be utilized to characterize such a motor syndrome.

Nowadays, some aspects of the clinical diagnosis of Parkinson’s disease are often deemed unsatisfactory [5] since the diagnostic process is considered to largely rely on clinical criteria, with the consequence that it is very difficult to formulate an early PD diagnosis, as well as an accurate and timely differential diagnosis between PD and other Parkinsonisms [5]. Moreover, other widely reported shortcomings are represented by the time-limited duration of the above-mentioned clinical examinations that may typically fail to capture daily fluctuations in motor signs from the presence of subjective aspects in the clinical ratings, and the circumstance that patients self-reporting is not always reliable [6,7].

To reduce these drawbacks, a great effort is being made to search for reliable markers/biomarkers and tools for early diagnosis and prognosis in PD [8]. Proposed biomarkers include clinical, imaging, biofluidic-base, and inflammation-related biomarkers for preclinical, prodromal, and clinical stages [9,10]. Some of the proposed tools and methods for the early detection of PD are based on analysing voice disorders [11,12], handwriting [13], olfactory testing [14], and accelerometery data [15]. Other proposed solutions based on the use of Artificial Intelligence include convolutional neural networks for eye tracking and facial expression analysis [16], Machine Learning-assisted speech analysis [17], and deep learning models for various modalities such as brain analysis and motion symptoms [18].

However, there are currently no means to identify prodromal PD with 100% certainty [19], neither standardized international criteria supporting PD diagnosis at a preclinical stage [10] nor confirmed biomarkers to provide early detection of PD efficiently [11,20]. On the other hand, an increasing number of studies have revealed that a combination of biomarkers can improve the diagnostic accuracy of individual biomarkers [9,10].

Digital biomarkers, smartphones, and smartwatches could provide objective, sensitive, real-world measures of PD, whereas continuous monitoring is considered a promising candidate marker for the detection of prodromal PD [21]. Indeed, several technology-based objective measures (TOMs) have already been proposed for the assessment of motor signs due to PD [22,23,24,25,26,27,28,29] and the extraction of key features in order to differentiate individuals with early PD from healthy controls [15,30,31].

In a recent study, a wrist-worn accelerometer was used to detect motor activity during a period of seven days, discovering that accelerometery data predicts prodromal PD since daily average acceleration in healthy subjects is greater than one in PD patients, and acceleration is reduced several years before PD diagnosis [15].

Another study found that wrist monitors are likely to overestimate steps and activity, particularly in people with tremors and dyskinesias. This is likely a consequence of these impairments, resulting in heightened upper limb movements which are mistakenly detected and recorded as steps and activity counts by the activity monitor [32]. As a consequence, only evaluating the motor activity of data measured by sensors could not be enough to reasonably distinguish healthy people from PD patients with mild to moderate tremors due to the concurrence of two opposite effects, the acceleration reduction due to PD, and possible overestimation of activity due to tremor.

Here, we present a new tool and method based on using a wrist-worn sensor to objectively measure characteristic motor features helping in supporting and facilitating the clinical diagnosis of Parkinsonian motor syndromes and PD. Moreover, we preliminarily investigate the use of another motor index to be used in combination with motor activity in order to refine distinguishing healthy people from PD patients with tremors. As reported in previous studies carried out with PD patients, such motor index is computed by the “PD-Watch” tool and is highly related to the severity and duration of tremors at rest [25,26,27]. Specifically, the primary distinction in computing the motor sings and the tremor index compared to other proposed methods lies in PD-Watch’s ability to not only assess frequency data from multi-axial sensors but also to recognize specific movement patterns typically associated with motor symptoms. For example, PD hand tremors typically occur between 3 Hz and 7 Hz with a supination–pronation characteristic, while tremor in patients with essential tremor (ET) typically occurs between 3 Hz and 12 Hz with a flexion–extension pattern [3]. Consequently, when detecting tremors at rest, the PD-Watch verifies if the movement frequency falls within the typical range mentioned above and if it exhibits a supination–pronation pattern. This approach helps to minimize the risk of misinterpretation by distinguishing characteristic tremor-at-rest motion from other physiological or pathological movements occurring at the same frequency as PD tremors but with a different movement pattern.

## 2. Materials and Methods

In this study, forty-one healthy controls and fifty-nine PD patients were recruited from the Department of Neurosurgery and Neurology, Neurology Unit of the Hospital of Potenza “San Carlo”, Italy. Patients provided written informed consent prior to participating in the study. Before the study began, its protocol was reviewed and approved by the Ethics Committee of Basilicata “CEUR”, Italy.

The UPRDS scoring was performed by a movement disorders expert and with PD patients under medication; after neurological examination, the data acquisition of movements began.

The proposed method is based on two steps and the use of a wrist-worn unit, including a tri-axial accelerometer (measurement range: from −8 g to 8 g), a battery, memory support, and a microcontroller unit. Data from the tri-axial accelerometer was sampled with a frequency of 50 Hz.

The first step of the proposed method (i.e., passive continuous recording) relies on the processing of data collected via a wrist-worn tri-axial accelerometer during a continuous, long-term passive recording session, where the total duration of each recording session is in the order of hours (e.g., 16 h per day or 24 h per day). In the second step of the proposed method (i.e., UPDRS-based active tests), the detection of possible bradykinesia/slowness and rest tremors is carried out during the execution of some motor tests related to Part 3 of MDS-UPDRS.

Finally, to assess the possible effects of dyskinesia in motor activity overestimation, data from twelve PD patients with mild to moderate dyskinesia from previous study [27] were considered.

More details on the recruited subjects are reported in Table 1.

### 2.1. Passive Continuous Recording

In phase 1, after neurological examination, the passive continuous recording of movements began for 24 consecutive hours. PD patients were asked to wear the portable system on their most affected wrist for continuous recording of motor activity in each patient’s normal environment; controls worn the tool on the left wrist or on the right wrist according to an individual preference. 

The main operations for detecting motor signs in PD patients have already been reported in part in previous studies [25,26,27], and the main new aspects of the processing procedure in PD patients and in controls are summarised below. In the pre-processing phase, detected data were processed with a low-pass filter to perform the offset compensation of accelerometric signals and data from the entire acquisition was divided into equal time sequences. For each time sequence, Fast Fourier Transform (FFT) and Power Spectral Density (PSD) were computed for each axis of the acceleration signal and the root mean square value of the three axes.

As PD hand tremors usually occur between 3 Hz and 7 Hz with a supination–pronation characteristic [3], in the proposed processing method a tremor is determined for the time sequences where a pronation-supination movement determines a maximum value in the frequency ranges between 3 Hz and 7 Hz [25]; such pronation-supination detection may be based on checking the presence of a characteristic distribution of spectral energy among the various axes of the measurement system with an inter-axis comparison [25]. Then, for each time sequence where a tremor is detected, the ratio of the integral of the PSD between 3 Hz and 7 Hz and the total PSD is computed to assess the severity of the tremor. Essentially, the daily pattern of said ratio is highly related to the daily pattern of the tremor severity [25,26,27].

Finally, two parameters/indexes were considered for each 24-h recording session:

- *a_RMS_* is the average value of the root mean square acceleration of the whole recording session (i.e., average daily motor activity).
- *BL*, also referred to as the tremor index, represents the average value of tremors for the entire 24-h recording (i.e., average daily tremor) computed by calculating the mean value of the daily pattern of said PSD ratio.

### 2.2. UPDRS-Based Active Tests

In phase 2, MDS-UPDRS scoring was carried out during neurological examination. During the motor assessment with MDS-UPDRS, patients were asked to wear a portable wearable system, configured as a wristwatch. The duration of each motor task was set to 10 s; the first and last seconds of the recording session were excluded, obtaining an 8-s session for each motor task. Data acquisitions with the wearable system were carried out for the following items of the MDS-UPDRS: (a) 3.5 hand movements (bradykinesia); (b) 3.6 pronation-supination movements of hands (bradykinesia); (c) 3.15 postural tremor of the hands; (d) 3.17 rest tremor amplitude.

As detailed in Table 2, the following parameters/indexes were considered during the motor task recording sessions:

- *a_RMS_* is the average value of the root mean square acceleration of the whole recording session.
- *A_AVG_*, the average value of the values *A_x_*, *A_y_*, *A_z_* in the range between 3 Hz and 7 Hz, where *A_x_*, *A_y_*, *A_z_* are the Fast Fourier Transforms of the time-acceleration signals *a_x_*, *a_y_*, *a_z_* for the *x*, *y*, and *z* axis, respectively.
- *A_MAX_*, the maximum value of *A_x_*, *A_y_*, *A_z_* in the range between 3 Hz and 7 Hz.
- For each axis of the tri-axial accelerometer, the frequency peaks occurring in a specific frequency range *f_P,x_*, *f_P,y_*, *f_P,z_* and the amplitude *A_P,x_*, *A_P,y_*, *A_P,z_* of each peak were computed.

The choice of the frequency interval between 3 Hz and 7 Hz is based on the findings that PD hand tremors usually occur within this range [3].

### 2.3. Statistical Analysis

For each characteristic, data from the PD patient group was compared to the data of healthy controls using the Wilcoxon rank sum test; *p*-values lower than 0.05 were considered statistically significant. For each comparison where the sample size of both groups was less than or equal to 20, *U* values from the Mann-Whitney U test were considered; we concluded that there is a significant difference between the groups when the *U* value is less than or equal to the *U* critical value.

The area under the receiver operating characteristic curves (AUCs), F1-score, Kappa statistic, sensitivity, specificity, and accuracy metrics were used to preliminary evaluate the exploratory results related to the performances of the PD-Watch recordings in distinguishing PD patients from healthy controls.

## 3. Results

**Figure 1:**
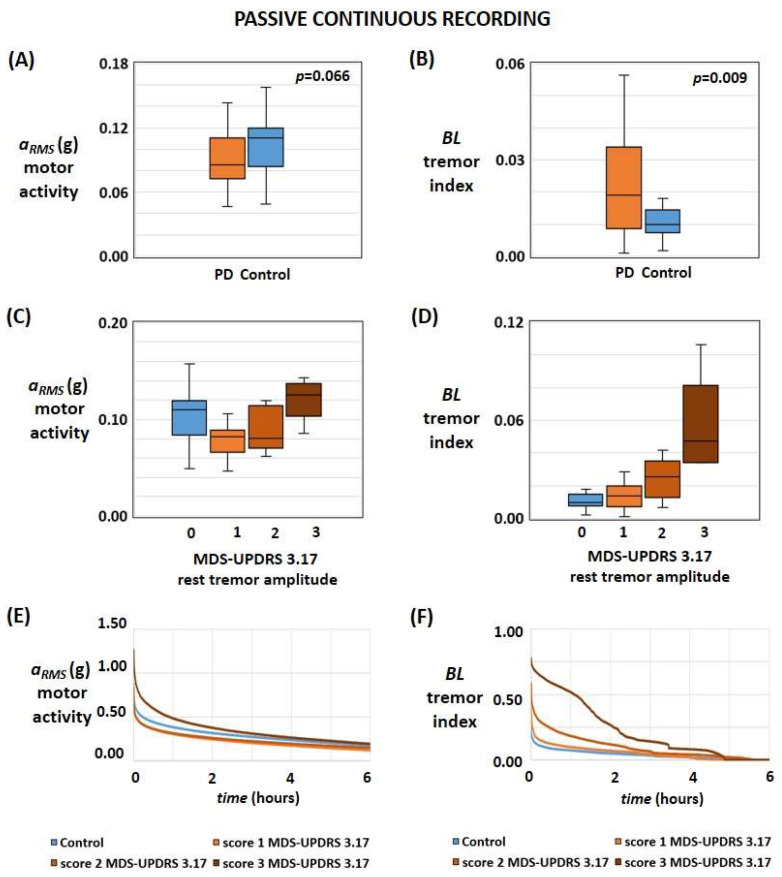
Results of passive continuous monitoring for each motor feature. (**A**,**B**) Box plot of motor activity (**A**) and tremor index (**B**) in healthy controls and PD patients. (**C**,**D**) Box plots of motor activity (**C**) and tremor index (**D**) in healthy subjects and PD patients with different severities of rest tremor. (**E**,**F**) The average temporal pattern of the motor activity (**E**) and tremor index (**F**) was determined during the 24-h recordings; for each recruited subject, values were sorted from the highest to the lowest value, and then the average value for each rest tremor severity was computed by considering all of the subjects with the same score of the item 3.17 of MDS-UPDRS.

**Figure 2:**
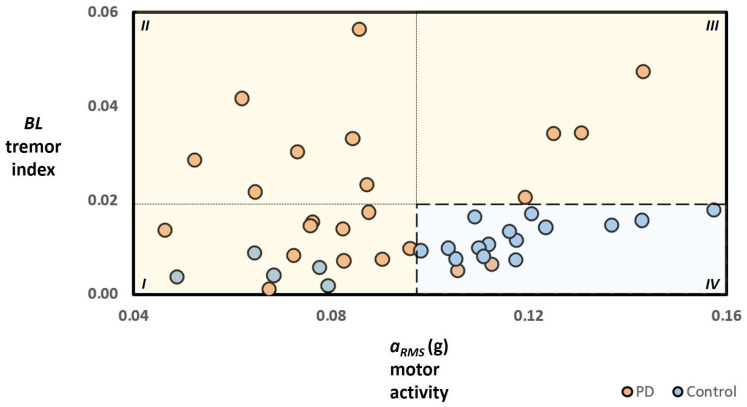
Results of passive continuous monitoring based on combination and concurrent use of both parameters—motor activity and tremor index—in discriminating controls and PD subjects. The areas labelled I, II, and III (orange background) reference the values of the motor activity and the tremor index corresponding to test results, where the determined motor status is associated with the presence of the Parkinsonian motor syndrome. In contrast, area IV (light blue background) refers to test results where the determined motor status is related to the absence of motor conditions assimilable to Parkinsonian motor syndrome. The dark orange dots over the light orange background represent a true positive; the dark orange dots over the light blue background represent a false negative; the dark blue dots over the light orange background represent a false positive; and the dark blue dots over the light blue background represent a true negative. It should be noted that data on one PD subject of area III is not shown for a merely illustrative reason.

**Figure 3:**
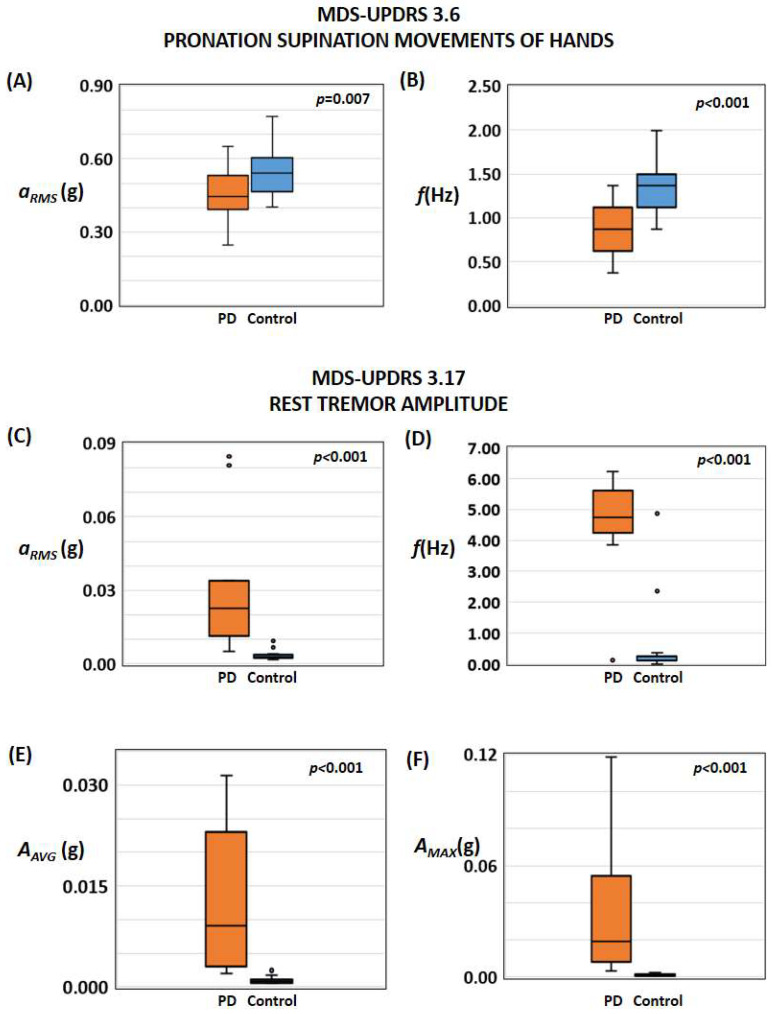
Results of active recording sessions. (**A**,**B**) Results related to the assessment of bradykinesia/slowness according to test 3.6 of the MDS-UPDRS executed with detecting the root mean square acceleration (**A**) of the tri-axial accelerometer and the frequency of the signal (**B**). (**C**–**F**) Results related to the assessment of wrist tremors at rest according to test 3.17 of the MDS-UPDRS executed with the detection of root mean square acceleration (**C**) of the tri-axial accelerometer, the frequency of the signal (**D**), the average value (**E**) and the maximum value (**F**) of the Fast Fourier Transforms of the acceleration signals between 3 Hz and 7 Hz.

**Figure 4:**
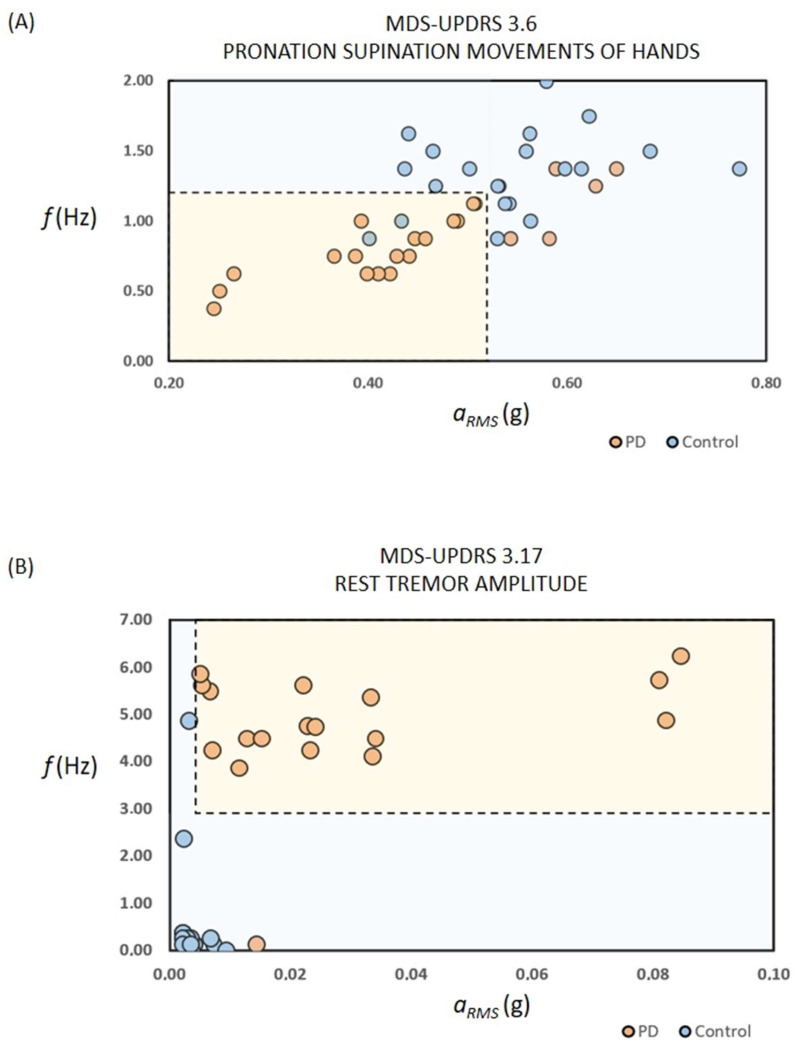
Results of active recording sessions with combination of root mean square acceleration and the signal’s frequency. (**A,B**) Results on assessment of bradykinesia/slowness (**A**) and rest tremors (**B**) based on a combination and concurrent use of the tri-axial accelerometer’s root mean square acceleration and the signal’s frequency. It should be noted that data on one PD subject with tremor is not shown for a merely illustrative reason.

**Figure 5:**
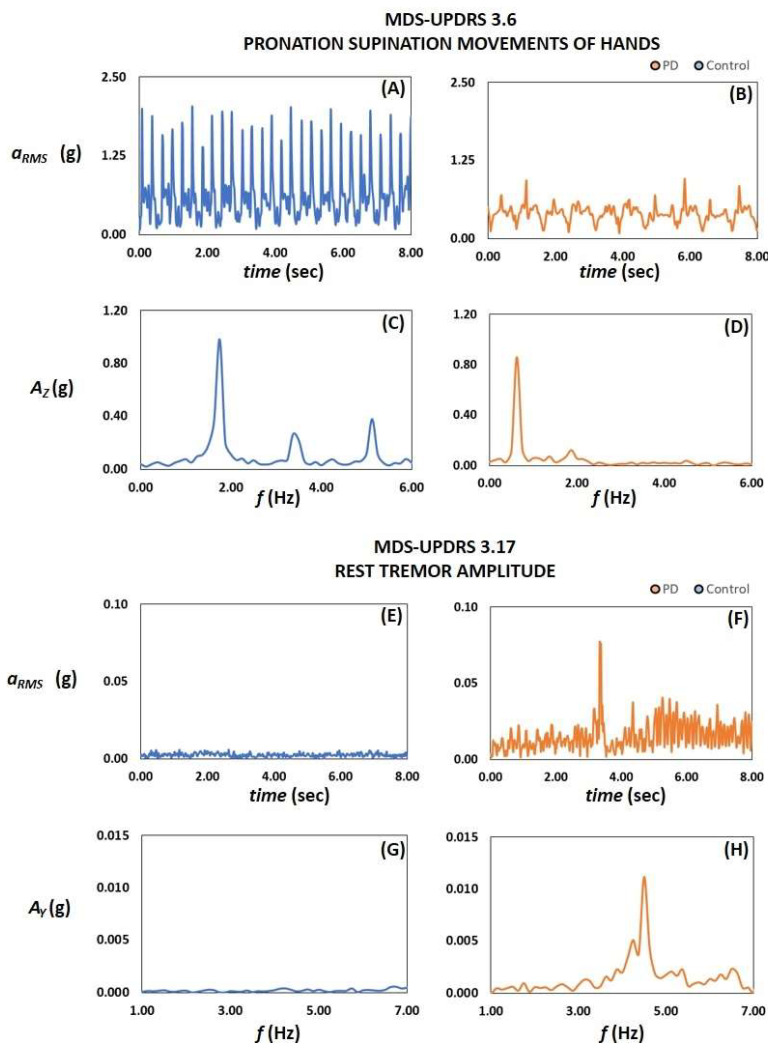
Active test. Temporal patterns of the motor activity a_RMS_ and the Fast Fourier Transform of the axial acceleration signals determined during the execution of motor tests 3.6 on pronation-supination (**A**–**D**) and test 3.17 on rest tremor amplitude (**E**–**H**) in a normal subject (blue) and a subject with PD (orange).

Detailed characteristics of the recruited subjects for each phase (i.e., passive recording and active testing) are reported Table 1 and in the Supplementary Materials File. The results and the outcome of the statistical analysis are summarised in Table 2 and Table 3. Figure 1, Figure 2, and Figure S1 show the results related to the passive continuous recording. Figure 3, Figure 4, Figure 5, Figure S2 and Figure S3 illustrate the results of the active tests. All relevant data is within the manuscript and its Supplementary Materials File.

The two phases were conducted sequentially, with patients from one phase being distinct from those in the other.

### 3.1. Passive Continuous Recording

Data obtained during passive continuous recording sessions are available for 24 PD patients with a disease duration lower than five years at the first visit and 20 healthy controls of subset 1.

Figure 1A shows the box plots of the typical values of *a_RMS_* in healthy subjects and PD patients; as reported in Table 2 and Table 3, even if the *p*-value is slightly greater than the statistically significant threshold (i.e., *p* = 0.066), the magnitude of the motor activity *a_RMS_*, as detected by the PD-Watch, seems smaller in PD than controls with the AUC metric equal to 0.626. On the other hand, the extent of the tremor index *BL*, as detected by the PD-Watch, was smaller in controls than in PD (AUC = 0.701; *p* = 0.009; Figure 1B). The box plots in Figure 1C illustrate the typical values of *a_RMS_* in healthy subjects and PD patients with different rest tremor severities, described by considering the score obtained for the item “3.17 rest tremor amplitude” of the MDS-UPDRS. As summarized in Table 2 and Table 3, statistical analysis conducted on the recruited subjects illustrates that the magnitude of the motor activity *a_RMS_* was higher in controls than in PD with slight tremors at rest (score 1 at item 3.17 of MDS-UPDRS). In contrast, motor activity *a_RMS_* did not significantly differ between controls and PD patients with mild-to-moderate tremor rest (score 2 to 3 at item 3.17 of MDS-UPDRS).

Similarly, the extent of the tremor index *BL* was smaller in controls than in PD with mild-to-moderate tremor rest and did not significantly differ between controls and PD patients with slight tremor rest (Figure 1D). Finally, Figure 1E,F show the average temporal values of the motor activity *a_RMS_* and the tremor index *BL* determined during the 24-h recordings for all recruited subjects, where values are sorted from the highest to the lowest value. Table 2 and Table 3 also report the statistical analysis of the motor activity *a_RMS_* and the tremor index *BL* and their respective performances (e.g., accuracy and F1-score, sensitivity, specificity, kappa statistic) in separating and distinguishing the two groups. Such tables and Figure 2 also illustrate the capabilities of the proposed method based on the combination and concurrent use of both parameters in discriminating controls and PD; in particular, these results illustrate that the combination of the two characteristics (i.e., the motor activity *a_RMS_* and the tremor index *BL*) improves the performances and accuracy with respect than those obtained by using just one parameter (e.g., accuracy is up to 73% if just one index is considered and raises to 84% by considering both parameters, whereas Cohen’s kappa coefficient raises from 0.48 to 0.68). Indeed, according to the proposed tool and method, subjects with higher motor activity and lower tremor index are classified as healthy, limiting the occurrence of False Negative cases related to high motor activity due to mild-to-moderate tremor at rest.

As shown in Figure 1D, the standard deviation of the *BL* index for PD patients with moderate tremor at rest (i.e., score 3 on the MDS-UPDRS 3.17) is higher than that for patients with slight to mild tremor (i.e., score 1 and 2 on the MDS-UPDRS 3.17). This is likely due to the fact that score 3 on item 3.17 of the MDS-UPDRS encompasses a wider range of resting tremor amplitude compared to scores 1 and 2 (i.e., slight and mild tremor corresponds to a maximal amplitude of tremor at rest lower than 1 cm and 3 cm, respectively, whereas moderate tremor refers to amplitude between 3 and 10 cm). Similarly, in the Figure 1B, the standard deviation of the tremor index *BL* for PD patients is greater than that of the controls.

Figure S1 of the Supplementary Materials File shows the box plots with the typical values of *a_RMS_* in healthy subjects and in further twelve PD patients with mild-to-moderate dyskinesia.

### 3.2. UPDRS-Based Active Tests

Data obtained during active sessions is available for 23 PD patients (of which 19 experience rest tremors) and 21 healthy controls of subset 1.

With reference to the test “3.5 hand movements”, the determined frequency and motor activity did not differ between controls and PD. On the other hand, during the test “3.6 pronation-supination movements of hands” related to slowness/bradykinesia assessment, the magnitude of the motor activity *a_RMS_* and the movement frequency *f*, as detected by the PD-Watch, were smaller in PD than controls (for *a_RMS_*: *p* = 0.007, AUC = 0.670, Figure 3A; for *f*: *p* < 0.001, AUC = 0.764, Figure 3B). Figure 4A and Table 3 also illustrate the effects of the combination and concurrent use of both parameters *a_RMS_* and *f* in discriminating controls and PD during test 3.6; according to the statistical analysis, such combination slightly increases the accuracy of the detection, whereas the Cohen’s kappa coefficient raises from 0.54 to 0.64.

Regarding the active test on rest tremors (3.17 rest tremor amplitude), the magnitude of the motor activity *a_RMS_*, the mean *A_AVG_*, and maximum *A_AVG_* values of the FFT of the acceleration signals in the range between 3 Hz and 7 Hz were higher in PD than controls (Figure 3C,E,F). Figure 4B and Table 3 also illustrate the effects of the combination and concurrent use of both parameters *a_RMS_* and *f* in discriminating controls and PD during test 3.17, where such combination did not significantly modify the detection performances. Comparable results were found for the active test on the postural tremor of hands 3.15 (box plot not shown).

As shown in Figure 3C–F, the standard deviation for PD patients is higher than that for controls, and this is likely due to the fact that scores of 1-3 on item 3.17 of the MDS-UPDRS encompass a wide range of tremor amplitude compared to controls (i.e., maximal amplitude of tremor at rest up to 10 cm versus no tremor).

Figure 5 illustrates the temporal patterns of the motor activity *a_RMS_* and of the Fast Fourier Transform of the axial acceleration signals determined during the execution of the motor tests on pronation-supination (3.6) and on rest tremors (3.17) in a normal subject and a subject with PD.

Figures S2 and S3 of the Supplementary Materials File report the typical values of *a_RMS_* and *f*, as detected during the tests 3.6 and 3.17, in healthy subjects and PD patients for different rest tremor and bradykinesia severities.

## 4. Discussion

To date, clinical expert opinion is the gold standard diagnostic technique for PD diagnosis, and there are currently no means to identify prodromal PD with 100% certainty. On the other hand, an increasing number of studies have revealed that a combination of biomarkers can improve the diagnostic accuracy of individual biomarkers.

The proposed tool combines motor characteristics and a two-step process (i.e., passive continuous recording and UPDRS-based active tests).

The first step of passive continuous recording may cover some complementary aspects of the active tests, aiming to reduce the issues related to the time-limited duration of the UPDRS-based motor tests with continuous monitoring and by considering a combination of various characteristic motor features. On the other hand, the second step on UPDRS-based active tests may keep a link with the current gold standards as much as possible since it concerns the objective quantification of two cardinal manifestations (i.e., bradykinesia and rest tremors) by executing some motor tests of Part 3 of the MDS-UPDRS with a wrist-worn accelerometer.

In the authors’ opinions, the main novel aspect of the proposed method relies on the passive continuous recording phase and the processed data based on the combination and concurrent use of multiple parameters for discriminating healthy subjects and people with Parkinsonian motor syndrome. As reported in the box plots in Figure 1C and Table 3, the magnitude of the motor activity *a_RMS_* was higher in controls than in PD, with slight tremor at rest, supporting the findings of a previous study, where the daily average acceleration in healthy subjects appeared greater than one in PD patients and acceleration was reduced several years before PD diagnosis [15]. However, according to the results of the present study, the motor activity *a_RMS_* may not significantly differ between controls and PD patients with mild-to-moderate rest tremors, supporting the use of motor activity *a_RMS_* as an individual biomarker might not be enough to accurately distinguish the two groups; indeed, as reported in another study [32], such conditions might be attributable to the overestimation of motor activity due to mild-to-moderate tremors. Thus, to refine distinguishing controls from subjects with Parkinsonian motor syndrome, we propose combining motor activity *a_RMS_* with another biomarker related to rest tremors (i.e., the tremor index *BL*).

According to the results obtained in the present study, even if the tremor index *BL* can differentiate the PD patients and controls enrolled in the study (AUC = 0.701; *p* = 0.009; Figure 1B), it did not significantly differ between controls and PD patients with slight tremor rest (Figure 1D). As reported in Figure 2 and Table 3, the combination of the two biomarkers (i.e., the motor activity *a_RMS_* and the tremor index *BL*) may improve the different capabilities with respect to the ones obtainable by using just one parameter (e.g., an accuracy increase from 73% to 84% by considering both parameters, whereas Cohen’s kappa coefficient raises to 0.68 corresponding to a substantial agreement). This may be due to the fact that such combination of biomarkers can allow one to take advantage of the complementary aspects of both parameters. Indeed, findings reveal that motor activity *a_RMS_* has a high capability in distinguishing controls from PD with slight tremors and may mitigate the poor performance of the tremor index *BL* with such subjects; nevertheless, the high capabilities of the tremor index *BL* in discriminating controls from PD with mild-to-moderate tremor may reduce the poor performance of the motor activity *a_RMS_* with such subjects. It should be noted that the motor activity of a subject is a global parameter whose final magnitude may be influenced by various factors (e.g., voluntary limb movement during normal daily life, steps and walking, pathological movements, and conditions such as tremors and dyskinesia, rigidity, postural instability). Therefore, the use of the tremor index *BL* might determine to separate from the global value of the motor activity the contribution of tremor, allowing to refine distinguishing healthy people from PD patients with tremors and reduce the occurrence of False Negative episodes.

Finally, even if the two steps of the proposed method are considered stand-alone processes, the UPDRS-based active tests may be considered as a step to get further confirmation of the results of the passive recording—or about the False Negative test results—by assessing the two cardinal manifestations (i.e., bradykinesia and rest tremors) by executing some motor tests according to the UPDRS scheme, representing a gold standard in the field of PD.

These findings demonstrate that combining said two digital biomarkers (i.e., motor activity and tremor at rest index) allowed controls to differ from early PD. Results are preliminary and exploratory, and other studies will be carried out to confirm these findings, taking into account the limitations of this study. In particular, possible improvements for future studies may include the recruitment of more PD patients and controls, the involvement of more medical centres, an increased number of PD experts, an extended duration of the passive recording period, execution of more active tests, the combination of passive and active tests for the same patient, the combination of more or other parameters during passive recording sequence, the comparison with other diagnostic tests and the examination of differential diagnosis performances in order to distinguish several types of Parkinsonism and essential tremor.

## 5. Conclusions

Early diagnosis of Parkinson’s disease remains challenging, and significant efforts are underway to identify reliable markers/biomarkers and tools for early diagnosis and prognosis. An increasing number of studies have shown that a combination of biomarkers can enhance diagnostic accuracy, with various promising candidate markers identified, including continuous monitoring.

This study proposes a new tool to support the diagnosis of Parkinsonian motor syndromes by processing data acquired through a wrist-worn sensor. Our research demonstrates the initial feasibility of this approach in differentiating controls from PD through passive continuous recording and/or UPDRS-based active tests. Specifically, our findings indicate that the execution of a passive, continuous recording session and the combination of two digital biomarkers (i.e., motor activity and rest tremor index) enabled differentiation between controls and early PD with an accuracy rate of 84%. Furthermore, UPDRS-based active tests may serve as a complementary step to validate the results of passive recording, reducing the occurrence of False Negative episodes and enhancing the overall performance of the proposed tool.

In conclusion, the proposed system and method are promising and suitable for integration into clinical trials and routine clinical practice to support the diagnosis of Parkinsonian motor syndromes.

## Figures and Tables

**Table 1:** Characteristics of the PD and control participants. With reference to the PD patients of active tests, it should be noted that only 19 of the 23 subjects had rest tremors. The symbol “//” means “not applicable”.

	Subset 1				Subset 2		Total (Subset 1 + Subset 2)	
	Continuous Recording		Active Test		Continuous Recording			
Characteristic	PD	Control	PD	Control	PD	Control	PD	Control
Number of subjects	24	20	23	21	12	0	59	41
Male	15	9	13	12	6	0	34	21
Female	9	11	10	9	6	0	25	20
Age								
Average (yr)	65	61	70	60	65	//	67	61
Standard deviation (yr)	6	4	4	6	3	//	5	6
Hoehn and Yahr Staging Scale								
Average	2.3	//	2.0	//	2.9	//	2.3	//
Standard deviation	0.8	//	0.9	//	0.8	//	0.9	//
MDS-UPDRS								
score 3.6 (pronation-supination)	1.9	//	1.8	//	1.5	//	//	//
score 3.17 (rest tremor)	1.7	//	1.7	//	1.1	//	//	//
score 4.1 (time with dyskinesia)	0.0	//	0.0	//	1.5	//	//	//
score 4.2 (impact of dyskinesia)	0.0	//	0.0	//	1.5	//	//	//

**Table 2:** Passive continuous recording. Results of the statistical analysis carried out with reference to each parameter in distinguishing healthy controls from PD subjects with different severity levels of tremors at rest.

Parameter	Class #1	Class #2	Number of PD	Number of Controls	Statistic	Outcome
motor activity *a_RMS_*	Control	PD	24	20	*p*-value	*p* = 0.066—not statistically significant
	Control	PD with MDS-UPDRS 3.17 score equal to 1	13	20	Mann-Whitney *U*	*U* = 49 < *U_C_*—statistically significant difference
	Control	PD with MDS-UPDRS 3.17 score equal to 2	6	20	Mann-Whitney *U*	*U* = 39 > *U_C_*—not statistically significant
	Control	PD with MDS-UPDRS 3.17 score equal to 2 and 3	11	20	Mann-Whitney *U*	*U* = 107 < *U_C_*—statistically significant difference
	Control	PD with mild-to-moderate dyskinesia	10	20	Mann-Whitney *U*	*U* = 80 > *U_C_*—not statistically significant
tremor index *BL*	Control	PD	24	20	*p*-value	*p* = 0.009—statistically significant difference
	Control	PD with MDS-UPDRS 3.17 score equal to 1	13	20	Mann-Whitney *U*	*U* = 109 > *U_C_*—not statistically significant
	Control	PD with MDS-UPDRS 3.17 score equal to 2	6	20	Mann-Whitney *U*	*U* = 21 < *U_C_*—statistically significant difference
	Control	PD with MDS-UPDRS 3.17 score equal to 2 and 3	11	20	Mann-Whitney *U*	*U* = 21 < *U_C_*—statistically significant difference

**Table 3:** Results on each parameter or each combination of more parameters in distinguishing controls from subjects with Parkinsonian motor syndrome. The following parameters are reference threshold: *a_T_*, *BL_T_*, *a_T_*_3_._6_, *f_T_*_1,3_._6_, *f_T_*_2,3_._6_, *a_T_*_,3_._17_, *A_T_*_1,3_._17_, *A_T_*_2,3_._17_, *A_T_*_3,3_._17_, *a_T_*_1,3_._15_, *a_T_*_2,3_._15_, *A_T_*_,3_._15_, *A_TM_*_,3_._15_. The symbol “//” means “not applicable”.

Test	Parameter(s)	Condition(s)	AUC	TP	TN	FP	FN	Sensitivity	Specificity	Accuracy	F1-Score	Kappa
Continuous passive recording	*a_RMS_*	*a_RMS_* < *a_T_*	0.626	17	15	5	7	0.71	0.75	0.73	0.74	0.45
	*BL*	*BL > BL_T_*	0.701	12	20	0	12	0.50	1.00	0.73	0.67	0.48
	*BL*, *a_RMS_*	(*a_RMS_* < *a_T_*) AND (*BL* > *BL_T_*)	//	22	15	5	2	0.92	0.75	0.84	0.86	0.68
Active test 3.6 Pronation-supination movements of hands	*a_RMS_*	*a_RMS_* < *a_T_*_,3_._6_	0.670	17	14	7	6	0.74	0.67	0.70	0.72	0.41
	*f*	*f* < *f_T1_*_,3_._6_	0.764	17	17	4	6	0.74	0.81	0.77	0.77	0.55
	*f*	*f* < *f_T2_*_,3_._6_	0.764	19	15	6	4	0.83	0.71	0.77	0.79	0.54
	*f*, *a_RMS_*	(*a_RMS_* < *a_T_*_,3_._6_) AND (*f* < *f_T_*_1,3_._6_)	//	17	19	2	6	0.74	0.90	0.82	0.81	0.64
Active test 3.17 Rest tremor amplitude	*a_RMS_*	*a_RMS_* > *a_T_*_,3_._17_	0.870	19	18	3	0	1.00	0.86	0.93	0.93	0.85
	*f*	3 Hz < *f* < 7 Hz	//	18	20	1	1	0.95	0.95	0.95	0.95	0.90
	*f*, *a_RMS_*	(*a_RMS_* > *a_T_*_,3_._17_) AND (3 Hz < *f* < 7 Hz)	//	18	21	0	1	0.95	1.00	0.98	0.97	0.95
	*A_AVG_*	*A_AVG_* > *A_T_*_1,3_._17_	0.919	19	19	2	0	1.00	0.90	0.95	0.95	0.90
	*A_AVG_*	*A_AVG_* > *A_T_*_2,3_._17_	0.919	18	20	1	1	0.95	0.95	0.95	0.95	0.90
	*A_MAX_*	*A_MAX_* > *A_T_*_,3_._17_	1.000	19	21	0	0	1.00	1.00	1.00	1.00	1.00
Active test 3.15 Postural tremor of hands	*a_RMS_*	*a_RMS_* > *a_T1_*_,3_._15_	0.803	17	17	4	2	0.89	0.81	0.85	0.85	0.70
	*a_RMS_*	*a_RMS_* > *a_T2_*_,3_._15_	0.803	16	18	3	3	0.84	0.86	0.85	0.84	0.70
	*f*	3 Hz < *f* < 7 Hz	//	12	20	1	7	0.63	0.95	0.80	0.75	0.59
	*f*, *a_RMS_*	(*a_RMS_* > *a_T1_*_,3_._17_) AND (3 Hz < *f* < 7 Hz)	//	11	21	0	8	0.58	1.00	0.80	0.73	0.59
	*f*, *a_RMS_*	(*a_RMS_* > *a_T2_*_,3_._17_) AND (3 Hz < *f* < 7 Hz)	//	11	21	0	8	0.58	1.00	0.80	0.73	0.59
	*A_AVG_*	*A_AVG_* > *A_T,3_._15_*	0.905	18	20	1	1	0.95	0.95	0.95	0.95	0.90
	*A_MAX_*	*A_MAX_* > *A_TM,3_._15_*	0.852	15	21	0	4	0.79	1.00	0.90	0.88	0.80

## Data Availability

All relevant data is within the manuscript and its Supplementary Materials.

## Supplementary Materials

The following supporting information can be downloaded at: https://www.mdpi.com/article/10.3390/s24061965/s1, Figure S1: Passive continuous recording including data on patients with dyskinesia. Figure S2: Active test on bradykinesia, together with the results of the regression model (truncated linear model). Figure S3: Active test on rest tremor amplitude (truncated exponential model).
