# Supplementary material for: A New Wrist-Worn Tool Supporting the Diagnosis of Parkinsonian Motor Syndromes
Source: Sensors (Basel). 2024 Mar 19;24(6):1965. doi: 10.3390/s24061965 (PMC10975887; doi:10.3390/s24061965)
Supplement: Supplementary file 1 [file sensors-24-01965-s001.zip › sensors-2892881-supplementary.pdf]

Supporting Information

The case-control study (without drugs) was conducted using one hundred subjects. The recruitment was conducted following a convenience sampling among patients from the Neurology Unit of the Hospital of Potenza “San Carlo”, Italy. Detailed characteristics of research participants are described in Table 1. Two subsets of subjects were considered; data from subset 1 was used to investigate and assess the performances of the proposed method for supporting and facilitating the diagnosis of Parkinsonian motor syndrome and PD and to evaluate the effect of tremors in the possible overestimation of motor activity in PD patients; data from subset 2 was used to explore the possible overestimation of motor activity on PD patients due to the presence of dyskinesia and motor fluctuations.

The following inclusion criteria were applied for the first subset of PD patients: a clinical diagnosis of PD; disease duration lower than 5 years at the first visit; bradykinesia; absence of motor fluctuations and/or dyskinesia; over 50 years of age; wearable system worn for at least 95% of the whole recording session. The recruited patients with slight-to-moderate bradykinesia.

The following inclusion criteria were applied for the second subset of PD patients: a clinical diagnosis of PD; tremors; bradykinesia; motor fluctuations and/or dyskinesia; over 50 years of age; wearable system worn for at least 95% of the whole recording session. The recruited patients were with mild-to-moderate bradykinesia.

Figure Captions

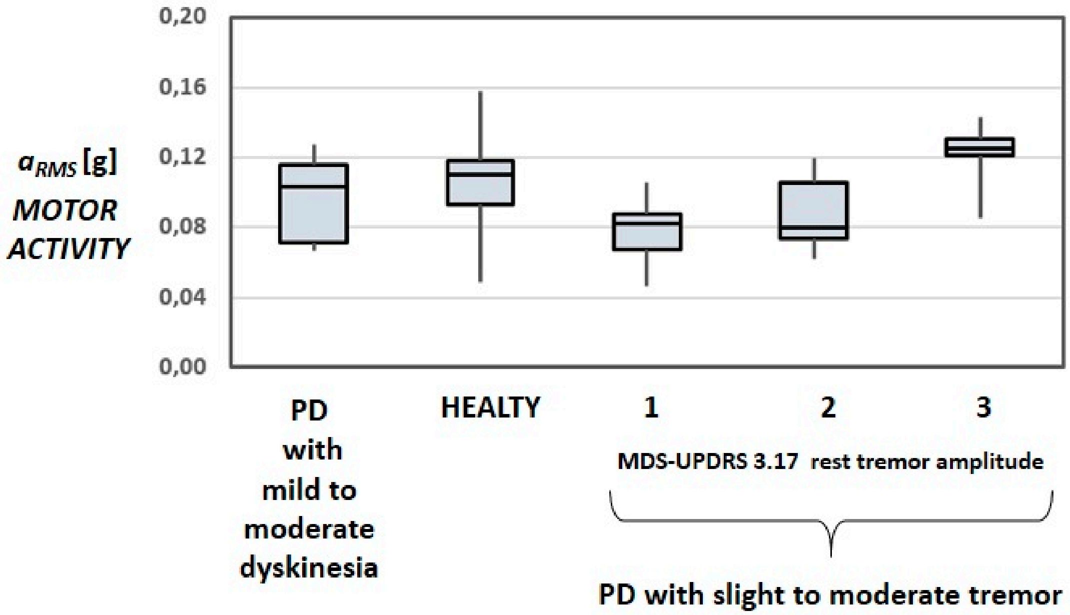

Figure S1. Passive continuous recording including data on patients with dyskinesia. Box plots with the typical values of a_{RMS} in healthy subjects in PD patients with tremors and without dyskinesia and in PD patients with mild-to-moderate

dyskinesia. Two patients with dyskinesia were excluded from this analysis (i.e., the wearable system was not worn for at least 95% of the whole duration of the recording sequence).

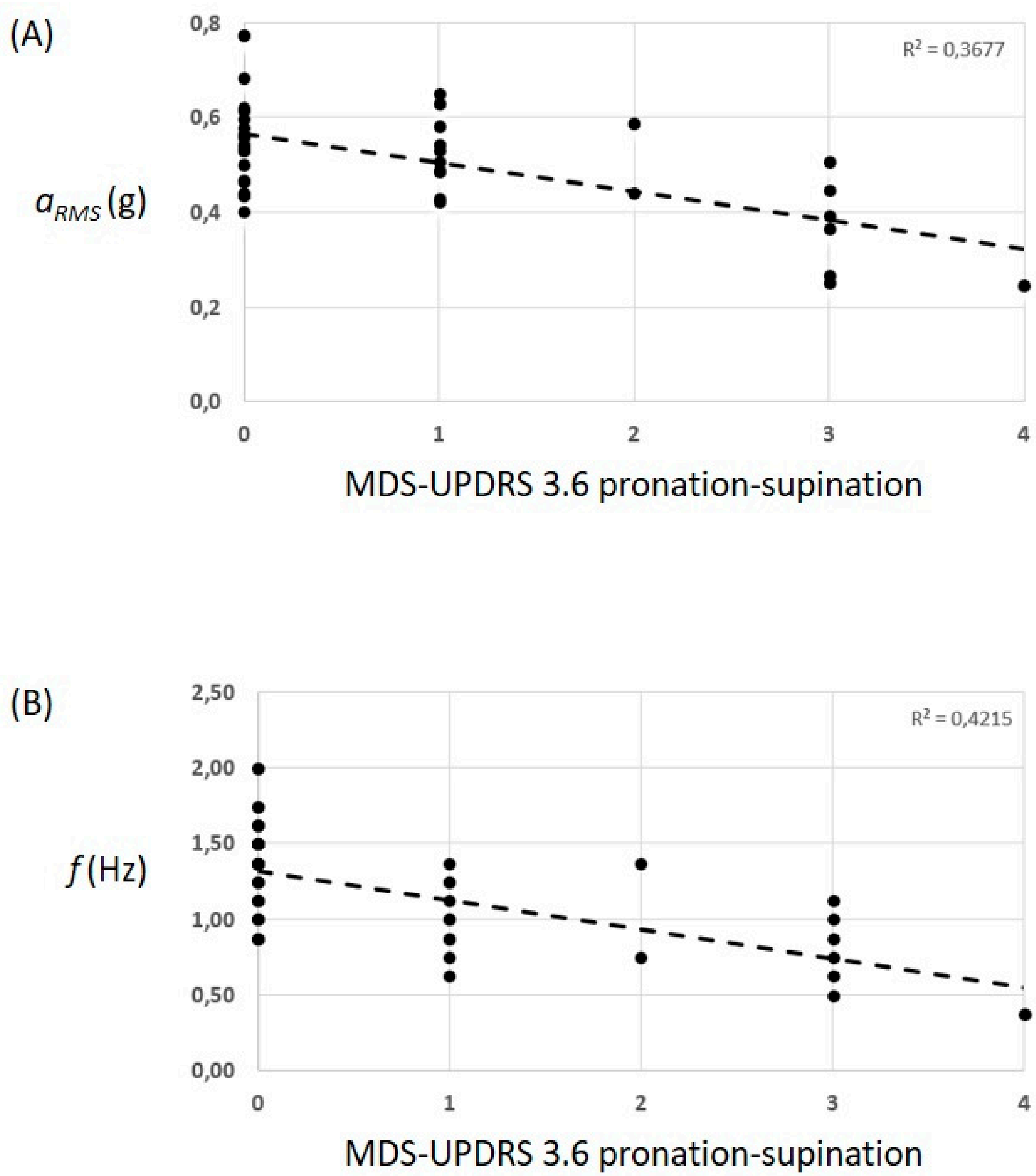

Figure S2. Active test on bradykinesia, together with the results of the regression model (truncated linear model). (a-b) Typical values of the root mean acceleration a_{RMS} (a) and of the frequency f of the signal (b) as detected during test 3.6, in healthy subjects and PD patients for different bradykinesia severities represented with the score at item 3.6 of the MDS-UPDRS.

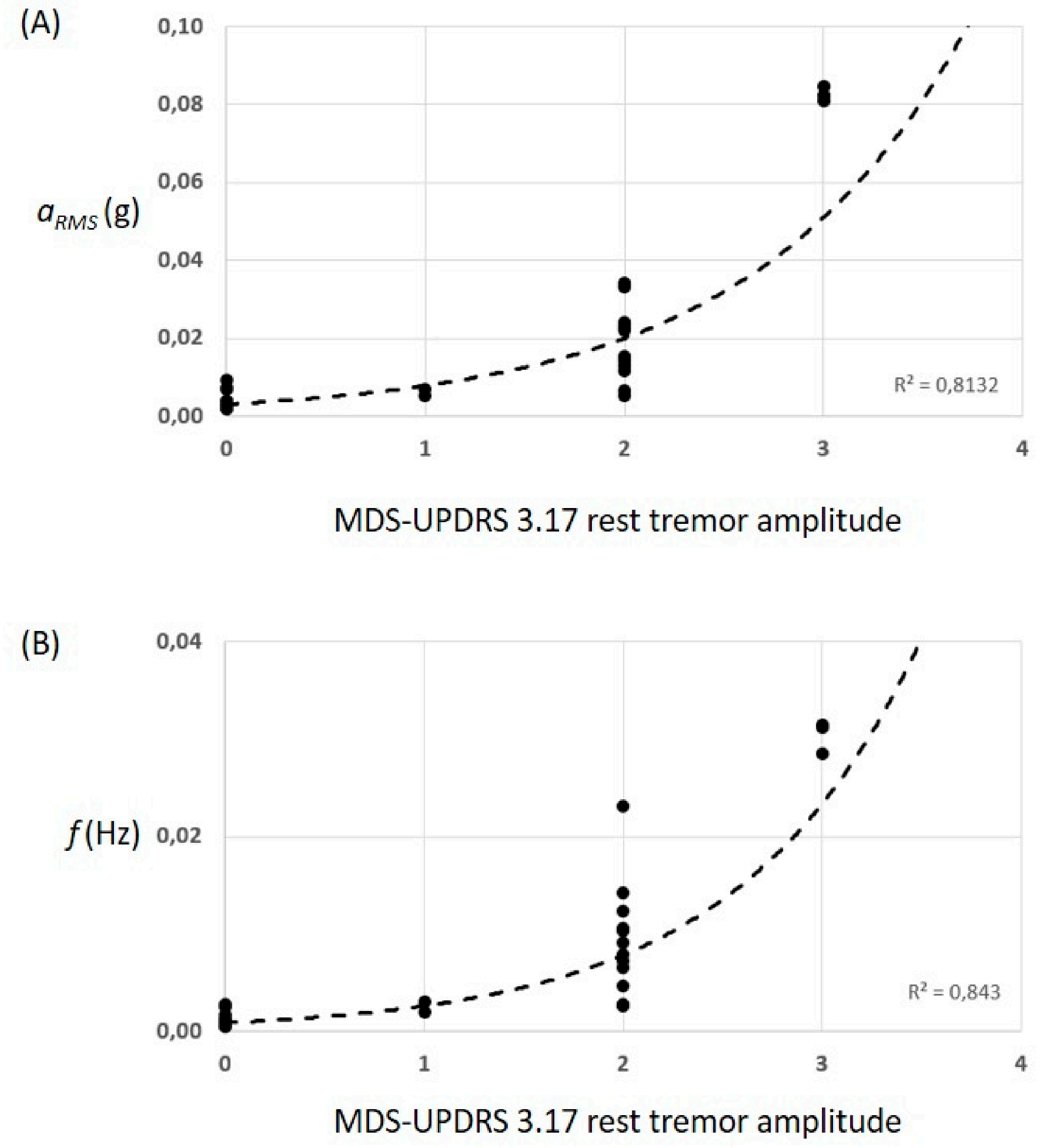

Figure S3. Active test on rest tremor amplitude (truncated exponential model). (a-B) Typical values of the root mean acceleration a_{RMS} (a) and mean values A_{AVG} (b) of the FFT of the acceleration signals between the range of 3 Hz and 7 Hz, as detected during test 3.17, in healthy subjects and PD patients for different of rest tremor severities represented with the score at item 3.17 of the MDS-UPDRS.
